# Effect of Delignification Process on the Properties of Barley-Straw Pulp and Paper

**DOI:** 10.3390/ma19183902

**Published:** 2026-09-14

**Authors:** Kateřina Hájková, Josef Bárta, Ida Skotnicová

**Affiliations:** 1 Faculty of Forestry and Wood Sciences, Czech University of Life Sciences Prague, Kamýcká 1176, 165 00 Prague, Czech Republic; hajkovakaterina@fld.czu.cz; 2 Institute of Natural and Synthetic Polymers, Faculty of Chemical and Food Technology, Slovak University of Technology in Bratislava, Radlinského 9, 812 37 Bratislava, Slovakia; ida.skotnicova@stuba.sk

**Keywords:** agricultural residue, peracetic acid, nitrate–alkaline delignification, soda pulping, Kappa number, barley straw, non-wood pulp, paper properties

## Abstract

**Highlights:**

• Barley straw was converted into pulp using three distinct delignification processes.
• Under the tested conditions, the peracetic-acid treatment gave the highest yield and the lowest Kappa number.
• The same treatment produced the highest tear index, burst index, and ISO brightness among the tested variants.
• Paper properties differed markedly despite relatively similar beating degrees.

**Abstract:**

Barley straw represents an underutilized agricultural residue with potential as a supplementary source of papermaking fibers. This study compared nitrate–alkaline, soda, and peracetic-acid delignification processes for the preparation of pulp from barley straw and evaluated the resulting laboratory paper sheets. The raw material was characterized by a cellulose content of 35.84%, an acid-insoluble lignin content of 18.79%, and ash content of 7.65%. Seven pulping variants were assessed in terms of total yield, reject content, Schöpper–Riegler degree, and Kappa number, while the paper sheets were tested for tear index, burst index, ISO brightness, Cobb_60_ water absorptiveness, and surface structure using optical microscopy. Total pulp yield ranged from 31.57% to 55.68%, and Kappa numbers varied from 13.50 to 47.86. Soda pulps had the highest Kappa numbers and the lowest brightness. Nitrate–alkaline treatments produced lower Kappa numbers than soda pulping but were generally accompanied by lower yields. Under the tested conditions, peracetic-acid delignification produced a pulp with a yield of 55.68%, a Kappa number of 13.50, an ISO brightness of 76.77%, a tear index of 6.26 mN·m^2^·g^−1^, and a burst index of 0.67 kPa·m^2^·g^−1^. Qualitative optical microscopy suggested a comparatively uniform fiber network. Among the evaluated variants, peracetic-acid delignification provided the most favorable combination of pulp yield, Kappa number, mechanical properties, and brightness; however, this finding is restricted to the specific treatment conditions applied in this study.

## 1. Introduction

The pulp and paper industry is increasingly seeking renewable alternatives to conventional wood-based raw materials. Growing demand for fiber-based products, competition for woody biomass, and the development of circular bioeconomy strategies have stimulated interest in agricultural residues as potential sources of papermaking fibers [1,2,3,4]. These materials are annually renewable, widely available, and generated within established agricultural production systems, allowing their valorization without establishing additional plantations solely for fiber production.

Cereal cultivation generates substantial quantities of straw composed mainly of cellulose, hemicelluloses, lignin, extractives, and inorganic constituents. Wheat, rice, barley, rye, and oat straws have consequently been investigated for the production of pulp, packaging materials, printing papers, handmade papers, and other cellulose-based products [2,3,4,5,6,7,8]. Compared with wood, cereal straws generally contain less lignin and can be delignified under relatively mild conditions. However, their shorter and morphologically heterogeneous fibers, together with higher contents of fines, ash, and silica, may negatively affect pulp drainage, chemical recovery, equipment operation, and selected mechanical properties of paper [3,4,9].

Barley (*Hordeum vulgare* L.) is an important cereal crop used primarily for animal feed, food production, and malting. Its cultivation is particularly relevant in the Czech Republic because of the country’s long-established malting and brewing industries. Spring malting barley represents a characteristic Czech agricultural and export commodity, and its production is closely associated with domestic malt and beer manufacture [10]. The cultivation of barley is accompanied by the annual generation of substantial quantities of straw, whose economic value remains considerably lower than that of the harvested grain. Material utilization of this residue could therefore complement the existing barley value chain and provide a locally available source of renewable fibers.

Barley straw typically contains approximately 30–45% cellulose, 20–30% hemicelluloses, and 12–20% lignin, although its composition varies according to cultivar, growing conditions, maturity, anatomical fraction, and analytical procedure [5,11,12]. Cellulose represents the principal fiber-forming component, whereas hemicelluloses contribute to fiber swelling and inter-fiber bonding. Lignin must be sufficiently removed or modified to allow fiber separation. Extractives, waxes, ash, and silica may additionally influence chemical consumption, pulp washing, drainage, and the surface properties of the resulting paper.

Previous studies have demonstrated that barley straw can be converted into papermaking pulp. Soda–anthraquinone pulping produced barley-straw pulp with relatively low residual lignin content and paper properties suitable for selected applications [5,13]. Alkaline pre-extraction followed by soda–anthraquinone pulping has also been investigated as a method for the integrated recovery of hemicelluloses and cellulose fibers from barley straw [14]. Barley straw has further been evaluated for ecological packaging, handmade paper, printing-paper formulations, and nanocellulose production [6,7,8,15]. Nevertheless, most studies have focused on a single pulping route or on blending barley-straw fibers with conventional wood pulps.

The properties of barley-straw pulp strongly depend on the selected delignification process and treatment severity. Soda pulping is commonly applied to agricultural residues because sodium hydroxide promotes fiber swelling, disrupts lignin–carbohydrate associations, and facilitates lignin dissolution without the use of sulfur-containing chemicals [1,5,13]. Excessive alkali concentration or treatment severity, however, may increase carbohydrate degradation, reduce pulp yield, and weaken the resulting fibers.

Nitrate–alkaline delignification is a two-stage process in which lignin is first oxidized and nitrated by nitric acid and subsequently dissolved during alkaline extraction. The method can produce comparatively bright, cellulose-rich fibrous material with a low residual lignin content and has been successfully applied to several non-wood feedstocks [16,17]. However, nitric acid consumption, corrosion risks, polysaccharide degradation, and the generation of nitrogen-containing effluents may limit its broader industrial application. The properties of the resulting pulp are also strongly affected by the acid and alkali concentrations and the severity of both treatment stages.

Peracetic acid is an oxidative delignifying agent that can be formed through the reaction of acetic acid with hydrogen peroxide. It promotes lignin removal and the oxidation of lignin-derived chromophores, thereby contributing to both delignification and brightness development [18,19,20]. Its selectivity toward lignin and carbohydrates depends on the reagent concentration, temperature, and treatment time. Although peracetic-acid treatment can reduce reliance on chlorine-containing bleaching chemicals, its practical application requires consideration of reagent stability, corrosion, process safety, chemical recovery, wastewater composition, and operating costs.

The suitability of pulp for papermaking cannot be evaluated solely based on pulp yield or residual lignin content. Mechanical strength, optical properties, and water absorptiveness are also affected by fiber length, flexibility, fines content, sheet density, and inter-fiber bonding. A comparative evaluation of chemically different delignification routes can therefore help identify, under specified experimental conditions, the treatment providing the most favorable balance between fiber liberation and paper performance.

Accordingly, the objective of this study was to evaluate barley straw as a locally available non-wood raw material for papermaking and to determine how different chemical delignification procedures affect the properties of the resulting pulp and paper. The raw material was chemically characterized and processed using nitrate–alkaline, soda, and peracetic-acid delignification. Laboratory sheets were subsequently evaluated in terms of tear index, burst index, ISO brightness, and Cobb_60_ water absorptiveness. The study aimed to compare the three delignification routes under the selected laboratory conditions and to identify the variant providing the most favorable combination of pulp yield, Kappa number, mechanical performance, optical quality, and water absorptiveness.

## 2. Materials and Methods

### 2.1. Raw Material

Barley straw (*Hordeum vulgare* L.) was obtained after harvest from an agricultural farm in Třebotov, Czech Republic. The material was stored under laboratory conditions until further processing. Before chemical analysis and pulping, the straw was manually sorted to remove residual grains, soil particles, and other impurities. The tested material consisted predominantly of stems, with a smaller proportion of leaves and spike residues.

For chemical characterization, a representative portion of the straw was reduced in size using an IKA Basic 10 knife mill (IKA-Werke GmbH & Co. KG, Staufen, Germany) according to TAPPI T 257. The milled material was homogenized and stored in sealed containers until analysis. All chemical analyses were performed in triplicate. The experimental design included chemical characterization of the raw material, seven pulping variants, preparation of laboratory paper sheets, determination of the Kappa number, physical and mechanical testing, and optical microscopy of the paper surface.

### 2.2. Chemical Characterization of Barley Straw

#### 2.2.1. Moisture Content

The moisture content of barley straw was determined according to TAPPI T 210. A representative sample was weighed and dried at 105 ± 2 °C until constant mass. After drying, the sample was cooled in a desiccator and reweighed. All subsequent chemical-composition results were expressed relative to the oven-dry mass of the raw material.

#### 2.2.2. Ash Content

Ash content was determined according to TAPPI T 211. A sample of known dry-matter content was placed in a pre-weighed crucible and incinerated in a muffle furnace at 525 ± 25 °C until constant mass. After cooling in a desiccator, the residual inorganic fraction was weighed and expressed as a percentage of the oven-dry sample mass.

#### 2.2.3. Extractives Content

Extractives were determined by Soxhlet extraction using an ethanol–toluene mixture at a volume ratio of 7:3. The extraction was performed for 6 h. After completion, the solvent was removed and the recovered extract was dried to constant mass. The extractives content was expressed relative to the oven-dry mass of the original sample.

The extractive-free solid residue was subsequently used for cellulose, holocellulose, and lignin determinations.

#### 2.2.4. Cellulose Content

Cellulose content was determined according to the Seifert method [21]. The extractive-free sample was treated under reflux with a mixture of acetylacetone, 1,4-dioxane, and hydrochloric acid. After the reaction, the suspension was cooled and diluted with methanol.

The isolated cellulose fraction was separated by filtration and successively washed with methanol, 1,4-dioxane, and hot distilled water. The residue was dried at 105 ± 2 °C until constant mass. The cellulose content was expressed as a percentage of the oven-dry sample mass.

#### 2.2.5. Acid-Insoluble Lignin

Acid-insoluble lignin was determined as Klason lignin according to TAPPI T 222. The extractive-free sample was hydrolyzed with concentrated sulfuric acid. The suspension was subsequently diluted to approximately 3% sulfuric acid and heated under reflux.

The insoluble residue was separated by filtration, washed with distilled water until neutral, and dried at 105 ± 2 °C until constant mass. The acid-insoluble lignin content was expressed relative to the oven-dry sample mass. Acid-soluble lignin was not determined.

#### 2.2.6. Holocellulose Content

Holocellulose content was determined according to the chlorite method developed by Wise et al. [22]. The extractive-free sample was treated with sodium chlorite under acidic conditions created by the addition of acetic acid. The reaction was conducted in a temperature-controlled water bath.

After delignification, the remaining polysaccharide fraction was separated by filtration, thoroughly washed with acetone, hot ethanol and cold distilled water, and dried at 105 ± 2 °C until constant mass. The holocellulose content was expressed as a percentage of the oven-dry sample mass.

Hemicellulose content was estimated as the difference between the independently determined holocellulose and cellulose contents. Consequently, hemicellulose was not measured directly, and the calculated value includes the combined analytical uncertainty of both gravimetric determinations.

### 2.3. Preparation for Barley Pulps

Seven experimental pulping variants representing three different delignification approaches were evaluated: nitrate–alkaline pulping, soda pulping, and peracetic-acid delignification. Each experimental variant was prepared as a single laboratory-scale pulping batch. Before pulping, the air-dried barley straw was manually cut into smaller segments to improve penetration of the cooking solutions.

Approximately 50 g of air-dried straw was used for each nitrate–alkaline and soda batch. After treatment, the cooked material was thoroughly washed with water, mechanically disintegrated using a laboratory pulper, and screened to remove incompletely cooked rejects. The accepted pulp and rejects were collected separately and dried to constant mass.

The experimental conditions applied to the individual pulp batches are summarized in Table 1.

Chemical concentration refers to the concentrations of the prepared treatment solutions.

The treatment conditions were selected as laboratory-scale reference conditions based on previously applied procedures for the individual delignification routes [13,16,17,18,19,20]. Because the three processes differ in reaction mechanism, chemical charge, number of treatment stages, and treatment time, the conditions were not intended to represent equivalent process severities or fully optimized industrial processes. The present study therefore compares the performance of the specific experimental variants under the applied conditions.

#### 2.3.1. Nitrate–Alkaline Pulping

For Nitrate–alkaline variants 1–4, barley straw was processed using a two-stage nitrate–alkaline treatment. In the first stage, the material was treated with a nitric acid solution for 30 min. A 6% HNO_3_ solution was used for all four variants.

The acidic treatment was followed by alkaline extraction with sodium hydroxide for an additional 30 min. NaOH concentrations of 10%, 15%, 25%, and 5% were used for Nitrate–alkaline variants 1, 2, 3, and 4, respectively.

After the alkaline stage, the treated material was mechanically disintegrated. The resulting pulp was neutralized using a 1% acetic acid solution and subsequently washed repeatedly with water until a neutral pH was reached.

#### 2.3.2. Soda Pulping

Soda pulping was performed as a single-stage alkaline treatment. Soda 1 was treated with a 15% NaOH solution at 101.7 °C for 30 min, whereas Soda 2 was treated with a 25% NaOH solution at 105.2 °C for 30 min.

The 30 min treatment time was intentionally selected as a relatively mild laboratory condition to evaluate the effects of two NaOH concentrations while limiting prolonged alkaline exposure of the carbohydrates.

After pulping, the material was thoroughly washed with water, mechanically disintegrated using a laboratory pulper (Lorenzen & Wettre, Stockholm, Sweden), and screened to remove incompletely cooked rejects.

#### 2.3.3. Peracetic-Acid Delignification

The oxidative cooking liquor was prepared by mixing glacial acetic acid with 30% hydrogen peroxide, previously cooled to approximately −2 °C, at a glacial acetic acid to hydrogen peroxide volume ratio of 7:3. The solution was prepared in advance and stored under dark and cool conditions to promote the formation of peracetic acid. The resulting cooking liquor contained approximately 8% peracetic acid and 6% residual hydrogen peroxide, and its preparation was adapted from the procedure reported by Barbash et al. [18].

Barley straw was mixed with the cooking liquor at a liquor-to-solid ratio of 10:1. Delignification was performed in a heat-resistant glass flask placed in a water bath at 95 °C. The flask was connected to a reflux condenser to minimize losses of volatile components and cooking chemicals during treatment.

The treatment was conducted for 45 min. After delignification, the cooked suspension was filtered through a screen and thoroughly washed with water to remove residual chemicals and dissolved reaction products.

The washed material was mechanically disintegrated in approximately 1500 mL of tap water using a laboratory pulper. The resulting fiber suspension was filtered through a screen, and excess water was removed manually before further processing.

This treatment is described as oxidative peracetic-acid delignification because peracetic acid was the principal active delignifying agent.

### 2.4. Determination of Pulp Yield and Reject Content

After pulping and screening of rejects, the produced pulp and incompletely cooked residues were collected separately and dried to constant mass. The screened pulp yield was calculated from the oven-dry mass of the accepted pulp relative to the oven-dry mass of the original barley straw.

The reject content was determined from the oven-dry mass of the incompletely cooked fraction relative to the initial oven-dry mass of the raw material. Total recovered solids were calculated as the sum of the screened pulp and reject fractions.

### 2.5. Determination of Schopper–Riegler Degree

The degree of beating of the prepared pulps was determined according to ISO 5267-1 using the Schopper–Riegler method. The results were expressed in degrees Schopper–Riegler (°SR).

### 2.6. Determination of Kappa Number

The extent of delignification and the amount of residual oxidizable material in the prepared pulps were assessed by determining the Kappa number according to ISO 302.

A defined oven-dry amount of disintegrated pulp was suspended in distilled water. Standardized potassium permanganate and sulfuric acid solutions were added, and the reaction was allowed to proceed for 10 min under continuous mixing.

After the reaction period, potassium iodide was added to reduce the remaining permanganate. The liberated iodine was titrated with a standardized sodium thiosulfate solution using starch as the endpoint indicator. A blank determination without pulp was performed under identical conditions.

The Kappa number was calculated from the amount of potassium permanganate consumed by the pulp and corrected according to the procedure specified in ISO 302.

### 2.7. Preparation of Laboratory Paper Sheets

Laboratory paper sheets were prepared from the obtained barley-straw pulps using a Rapid-Köthen sheet former (Birkenau, Germany) in accordance with ISO 5269-2. The pulp suspensions were dispersed in water and adjusted to produce sheets with a basis weight of approximately 65 g·m^−2^. The actual basis weight of the prepared sheets ranged from 62.58 to 71.34 g·m^−2^.

After formation, the wet sheets were pressed and dried under the standard conditions of the Rapid-Köthen procedure. Before further testing, all laboratory sheets were conditioned at 23 ± 1 °C and 50 ± 2% relative humidity in accordance with TAPPI T 402 sp-08.

For each pulp variant, ten laboratory sheets were prepared from the corresponding single batch. All the properties determined (tear index, burst index, ISO brightness and Cobb_60_ water absorption) were measured in ten replicates and subsequently converted to the basis weight of the individual sheets. The results were expressed as mean values ± standard deviations.

### 2.8. Physical, Mechanical, and Optical Properties of Paper Sheets

#### 2.8.1. Tear Index

Tear index was determined using the Elmendorf method according to ISO 1974. The paper specimens were clamped in the testing instrument and provided with a standardized initial cut. The pendulum was subsequently released, and the force required to propagate the tear through the remaining test length was recorded.

The results were expressed as tearing force in mN and as tear index in mN·m^2^·g^−1^, taking the basis weight of the tested sheets into account.

#### 2.8.2. Burst Index

Burst index was determined according to ISO 2758 using a Mullen-type bursting tester (FRANK- PTI, Birkenau, Germany). The paper specimen was clamped over an elastic diaphragm, and hydraulic pressure gradually increased until rupture occurred.

The maximum pressure recorded at rupture was expressed in kPa. The burst index was calculated by relating the bursting strength to the basis weight of the paper and was expressed in kPa·m^2^·g^−1^.

#### 2.8.3. ISO Brightness

The optical properties of the laboratory sheets were evaluated by measuring ISO brightness according to ISO 2470. Measurements were performed using a laboratory spectrophotometer Elrepho (Datacolor, Lawrenceville, NJ, USA).

A sufficient number of paper layers was used to prevent the background from affecting the measurement. The diffuse blue reflectance factor was measured at an effective wavelength of 457 nm, and the results were expressed as percentage ISO brightness.

#### 2.8.4. Water Absorptiveness

Water absorptiveness was determined using the Cobb method according to ISO 535. Pre-weighed paper specimens were exposed to distilled water from one side for 60 s.

After exposure, the water was removed, and excess surface moisture was eliminated using standardized blotting paper and a metal roller. The specimens were immediately reweighed, and the Cobb 60 value was calculated from the increase in specimen mass relative to the exposed surface area. The results were expressed in g·m^−2^.

### 2.9. Optical Microscopy

The surface structure and fiber arrangement of the laboratory paper sheets were examined using a Leica DVM6 M digital microscope (Leica Microsystems, Wetzlar, Germany).

Representative specimens were taken from each paper variant and placed directly on the microscope stage without coating or additional chemical preparation. Images were acquired under reflected-light conditions using identical illumination and magnification settings for all samples.

The microscopic images were used to qualitatively evaluate fiber separation, the presence of fiber bundles and incompletely delignified particles, the apparent occurrence of fine material, sheet homogeneity, visible open areas, and structural discontinuities within the paper network. No quantitative determination of fiber-length distribution, fines content, pore-size distribution, or network density was performed.

### 2.10. Statistical Analysis

Chemical analyses of the raw barley straw were performed in triplicate. For the paper properties, the number of sheet-level measurements per pulp variant was n = 10 for tear index, burst index, ISO brightness, and Cobb_60_ water absorptiveness. Differences among the seven pulp variants were evaluated separately for each property using one-way analysis of variance (ANOVA), followed by Tukey’s honestly significant difference (HSD) post hoc test at a significance level of α = 0.05. Results are presented as mean values ± standard deviations. Statistical analyses were performed using Statistica, version 14.1 (TIBCO Software Inc., Palo Alto, CA, USA). Each pulp variant was prepared as a single pulping batch; consequently, the measurements represent sheet-level replicates rather than independent pulping replicates. The statistical comparisons therefore describe differences among sheets produced from the specific experimental batches and do not quantify between-batch process variability.

## 3. Results

### 3.1. Chemical Composition of Barley Straw

The chemical composition of the barley straw is presented in Table 2. The initial moisture content of the air-dried material was 5.42 ± 0.64%. All remaining compositional parameters were expressed relative to the oven-dry mass of the raw material.

The analyzed barley straw was characterized by a relatively high ash content, which is typical of annual agricultural biomass. The cellulose and estimated hemicellulose contents indicated that structural polysaccharides constituted the major chemical fraction of the raw material.

### 3.2. General Properties of Barley-Straw Pulps

The general properties of the barley-straw pulps, including total yield and reject content, are summarized in Table 3.

Although the soda pulps showed relatively high total yields, their Kappa numbers remained higher than those of the peracetic-acid pulp. Under the tested conditions, the peracetic-acid variant provided both the highest total yield and the lowest Kappa number.

### 3.3. Mechanical, Optical, and Physical Properties of Laboratory Paper Sheets

**Figure 1:**
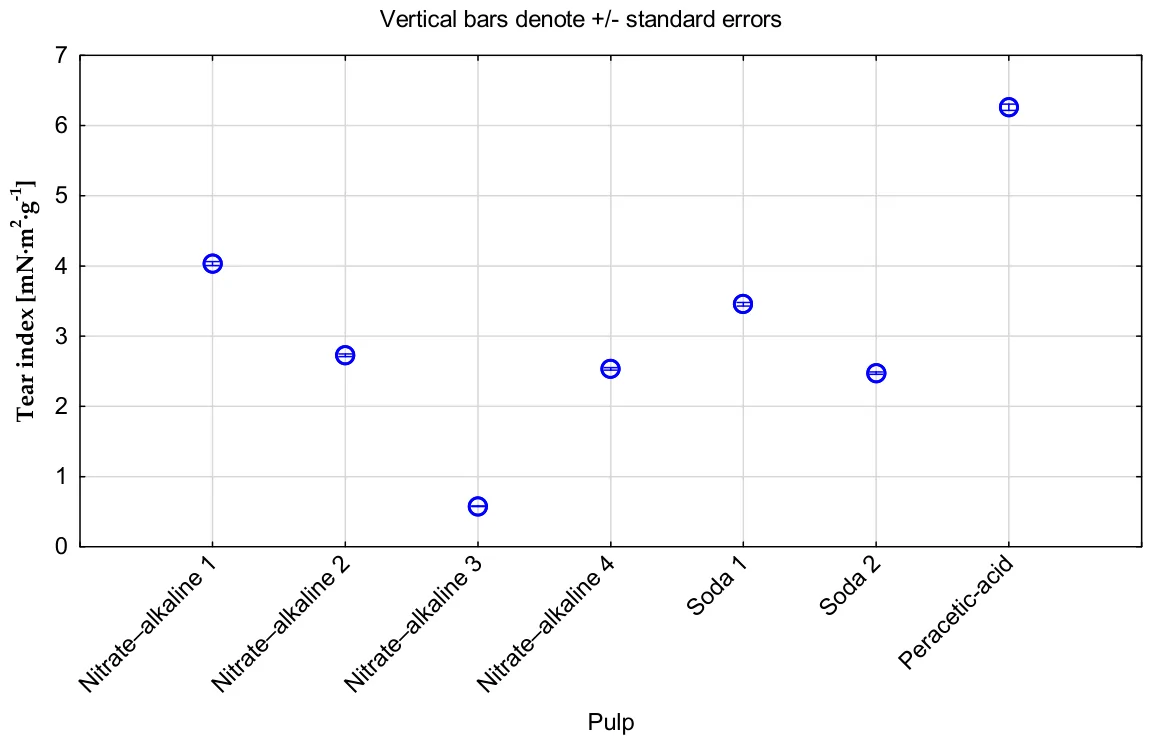
Tear index of laboratory paper sheets prepared from barley-straw pulps using different delignification treatments. Values are presented as means ± standard deviations (*n* = 10). One-way ANOVA: F(6,14) = 4640.1, *p* < 0.001 followed by Tukey’s HSD test (*p* < 0.05).

**Figure 2:**
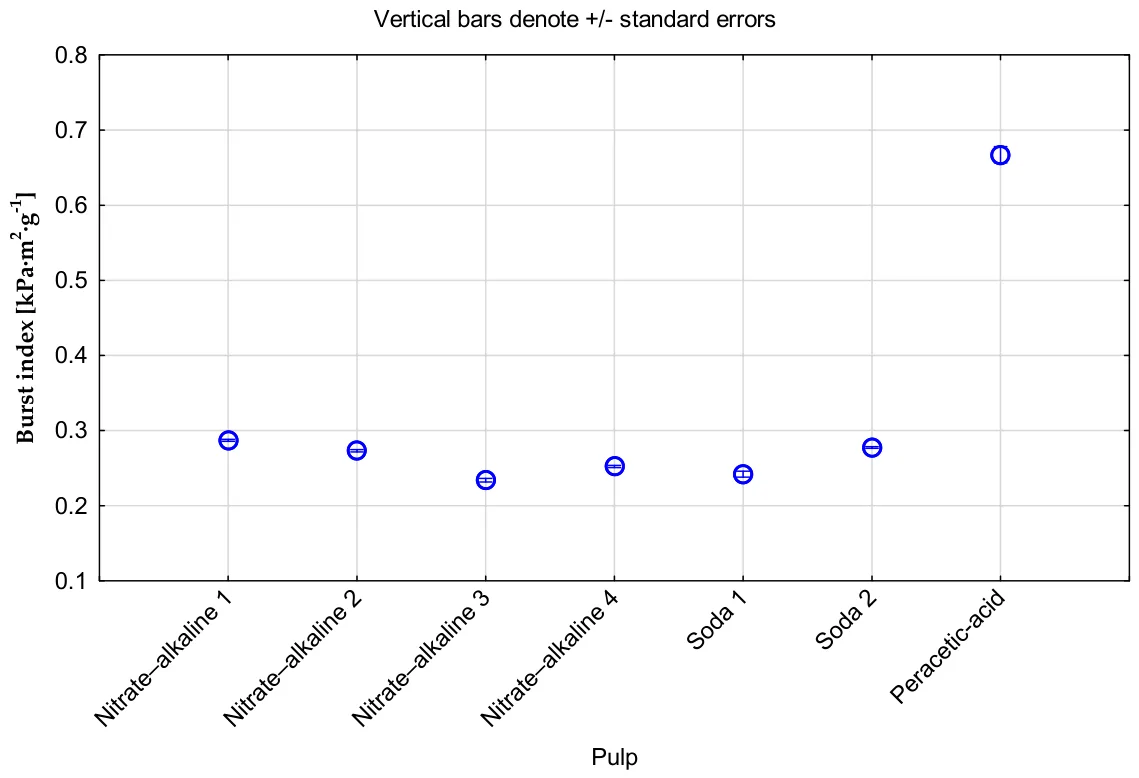
Burst index of laboratory paper sheets prepared from barley-straw pulps using different delignification treatments. Values are presented as means ± standard deviations (*n* = 10). One-way ANOVA: F(6,14) = 1151.0, *p* < 0.001 followed by Tukey’s HSD test (*p* < 0.05).

The target basis weight of the laboratory paper sheets was 65 g·m^−2^. The mean basis weight of the individual pulp variants ranged from 62.58 ± 3.38 to 71.34 ± 5.86 g·m^−2^. The mechanical, optical, and physical properties of the sheets prepared from the individual barley-straw pulps are summarized in Table 4. A graphical comparison of the tear and burst indexes is presented in Figure 1 and Figure 2.

One-way ANOVA identified significant differences among the seven sets of sheet-level measurements for tear index (F(6,14) = 4640.1, *p* < 0.001), burst index (F(6,14) = 1151.0, *p* < 0.001), ISO brightness (F(6,28) = 394.16, *p* < 0.001), and Cobb60 water absorptiveness (F(6,7) = 52.886, *p* < 0.001). Tukey’s HSD groupings are presented in Table 4. Because each pulp variant originated from a single pulping batch, these statistical results characterize differences among sheets produced from the tested batches rather than between-batch process variability.

In the sheet-level comparison, the peracetic-acid pulp exhibited significantly higher tear and burst indices than all tested variants. Its tear index reached 6.26 ± 0.08 mN·m^2^·g^−1^, while the burst index was 0.67 ± 0.02 kPa·m^2^·g^−1^. In contrast, the lowest tear and burst indices were recorded for Nitrate–alkaline 3, with values of 0.58 ± 0.01 mN·m^2^·g^−1^ and 0.23 ± 0.00 kPa·m^2^·g^−1^, respectively.

The ISO brightness of the laboratory sheets ranged from 28.77 ± 0.27% to 76.77 ± 1.38%. The highest brightness was obtained for the peracetic-acid pulp, which differed significantly from all other variants in the sheet-level comparison, whereas the lowest values were observed for the two soda pulps. Among the nitrate–alkaline variants, ISO brightness ranged from 48.59 ± 4.19% to 54.23 ± 1.50%.

The Cobb60 values ranged from 16.77 ± 0.74 to 41.67 ± 3.29 g·m^−2^. The lowest water absorptiveness was recorded for Soda 1 and Soda 2, while the highest values were measured for Nitrate–alkaline 3 and Nitrate–alkaline 4. The peracetic-acid pulp showed an intermediate Cobb60 value of 23.45 ± 2.09 g·m^−2^. According to Tukey’s HSD test, its Cobb_60_ value did not differ significantly from those of Nitrate–alkaline 1, Nitrate–alkaline 2, Soda 1, or Soda 2, but it was significantly lower than those of Nitrate–alkaline 3 and Nitrate–alkaline 4.

### 3.4. Optical Microscopy of Laboratory Paper Sheets

**Figure 3:**
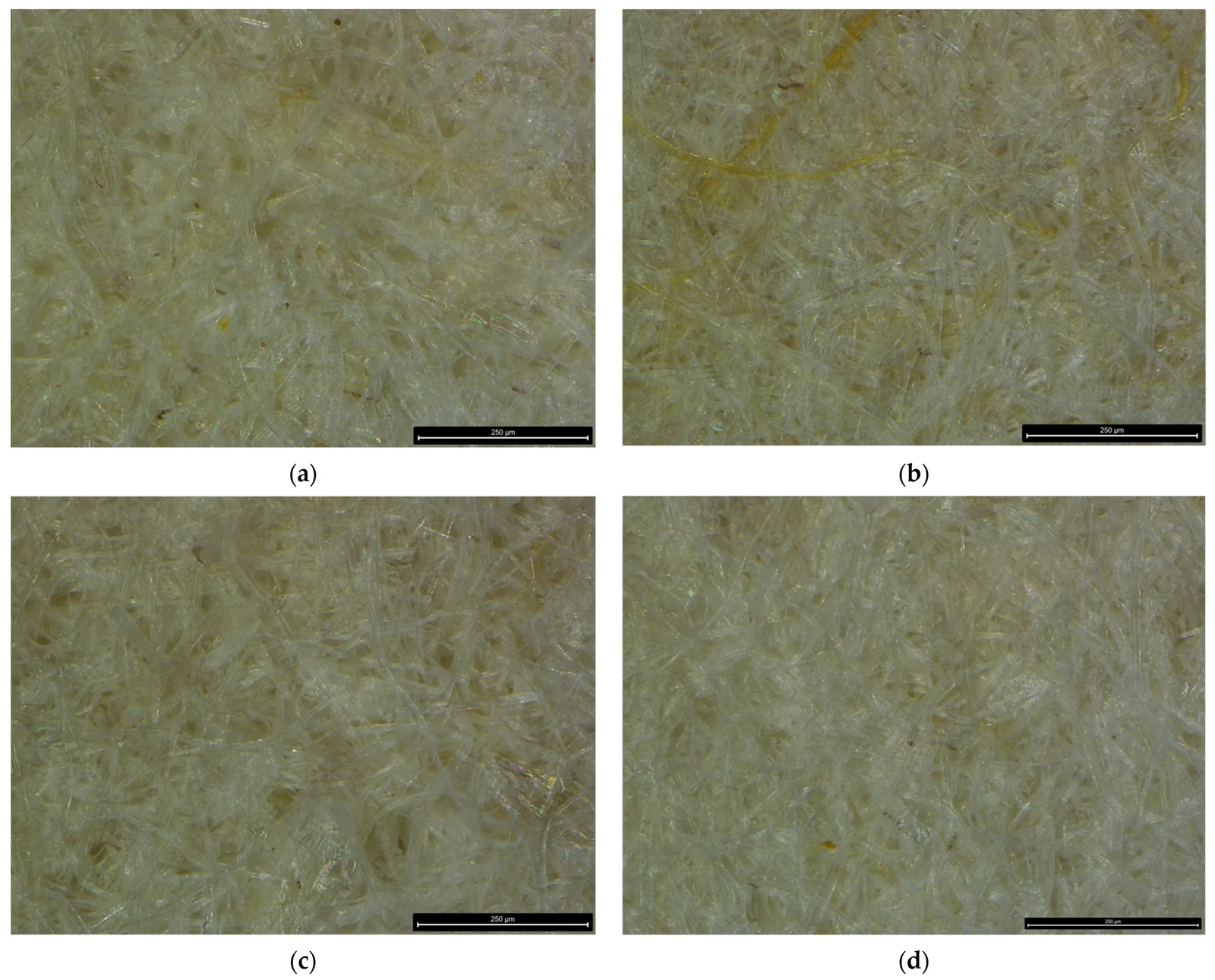
Representative optical micrographs of laboratory paper sheets prepared by nitrate–alkaline delignification: (**a**) Nitrate–alkaline 1; (**b**) Nitrate–alkaline 2; (**c**) Nitrate–alkaline 3; and (**d**) Nitrate–alkaline 4. Images were acquired at 1200× magnification using a Leica DVM6 M digital microscope. Scale bar: 250 µm.

**Figure 4:**
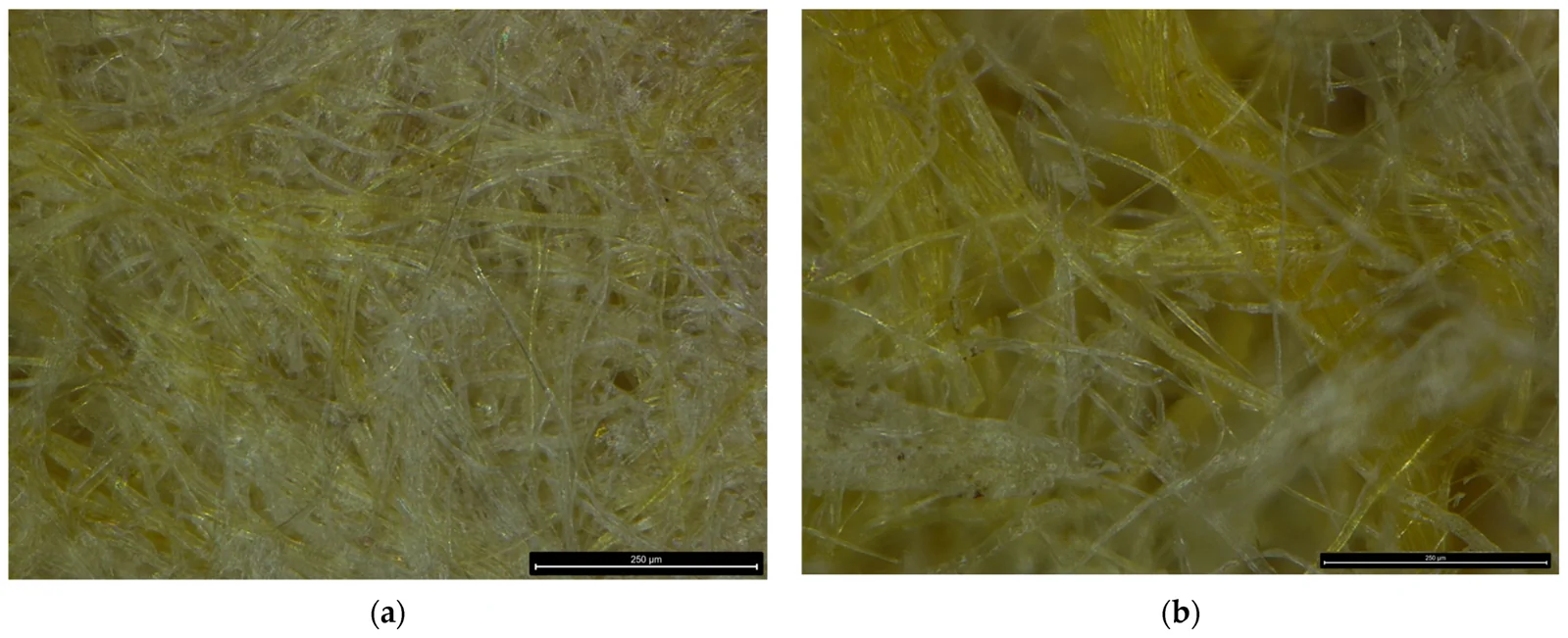
Representative optical micrographs of laboratory paper sheets prepared by soda pulping: (**a**) Soda 1 and (**b**) Soda 2. Images were acquired at 1200× magnification using a Leica DVM6 M digital microscope. Scale bar: 250 µm.

**Figure 5:**
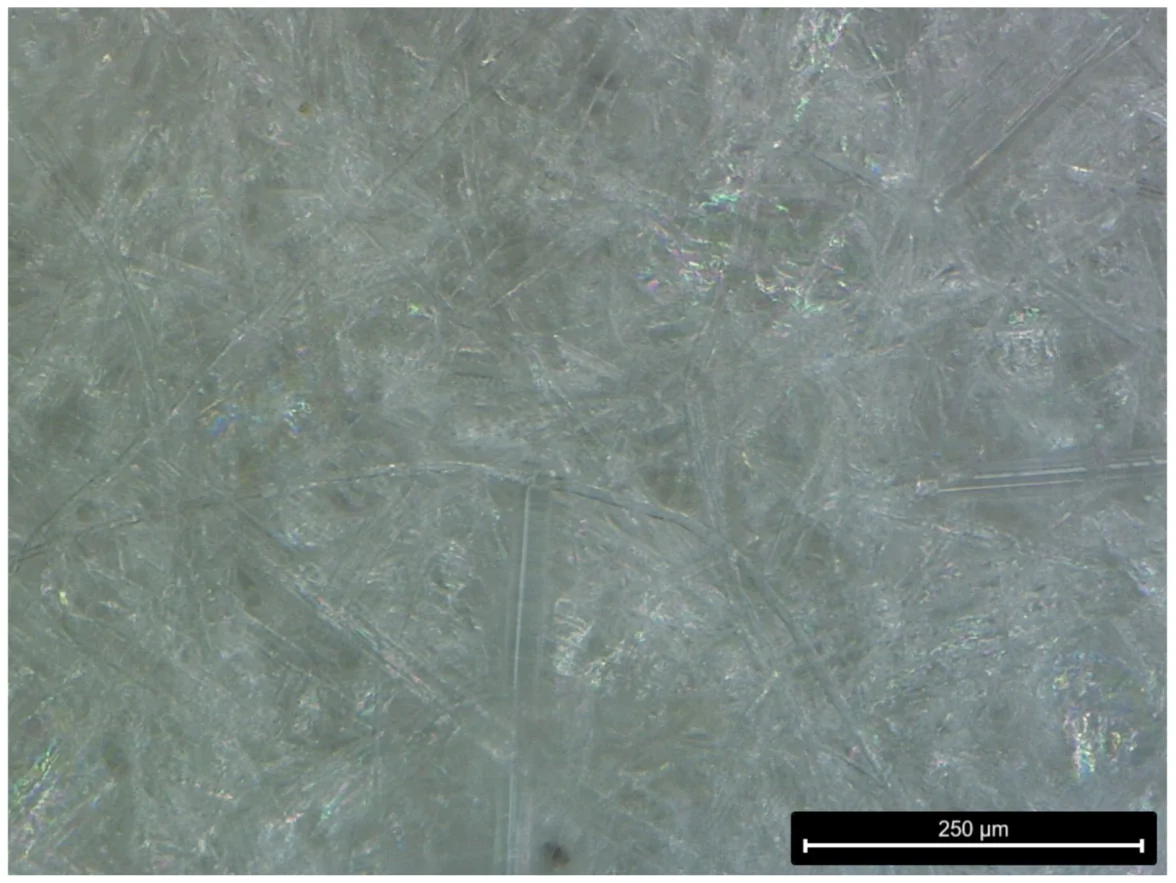
Representative optical micrograph of the laboratory paper sheet prepared by peracetic-acid delignification. The image was acquired at 1200× magnification using a Leica DVM6 M digital microscope. Scale bar: 250 µm.

Representative optical micrographs of the laboratory paper sheets are presented according to the applied delignification process. Figure 3 shows the four nitrate–alkaline variants, Figure 4 presents the two soda-pulp variants, and Figure 5 shows the sheet prepared from the peracetic-acid pulp. All images were acquired at 1200× magnification using a Leica DVM6 M digital microscope.

The nitrate–alkaline sheets exhibited visible differences in fiber separation, network homogeneity, and the occurrence of residual fiber bundles among the individual treatment variants. The soda-pulp sheets showed a relatively compact fiber network, although local structural irregularities and incompletely separated particles were still visible. The peracetic-acid sheet appeared to exhibit a comparatively uniform and continuous fiber network, with fewer visible coarse bundles and pronounced discontinuities. These observations are qualitative because fiber-length distribution, fines content, pore-size distribution, and network density were not measured quantitatively.

## 4. Discussion

The results show that barley straw can provide pulp suitable for paper production, but the final properties depend strongly on how selectively the raw material is delignified. The three processes tested in this study produced markedly different combinations of pulp yield, residual lignin, strength, brightness, and water absorptiveness. Among the specific experimental variants evaluated in this study, the peracetic-acid treatment provided the most favorable combination of pulp yield, Kappa number, brightness, and mechanical properties. However, because the three processes differed in chemical charge, treatment time, number of stages, and reaction mechanism, the results do not demonstrate the general superiority of peracetic-acid delignification over nitrate–alkaline or soda pulping. They apply specifically to the laboratory conditions investigated here, which were not designed to represent equivalent process severities or fully optimized industrial processes.

The chemical composition of barley straw, 35.84% cellulose, 40.77% hemicelluloses, 18.79% lignin, and 7.65% ash, was within the ranges previously reported for cereal straws [2,5]. The reported lignin value represents acid-insoluble lignin, whereas the hemicellulose content was calculated as the difference between holocellulose and cellulose determined by separate gravimetric procedures. Consequently, the hemicellulose value should be regarded as an operational estimate that incorporates the uncertainties of both analytical methods.

The relatively high ash content represents a potential disadvantage for industrial processing. Cereal-straw ash commonly contains silica and other inorganic components that may impair liquor clarification and washing, contribute to deposits and scaling, and complicate evaporation and chemical recovery [3,9]. However, the elemental and mineralogical composition of the ash was not determined in the present study; therefore, the specific effects of the barley-straw ash on chemical recovery cannot be quantified from these results. Appropriate feedstock cleaning, removal of soil contamination, and process-specific management of silica-rich streams would need to be considered during scale-up. The high hemicellulose content may contribute to fiber swelling and inter-fiber bonding, but its retention after pulping was not measured.

The pulp yields demonstrate substantial differences in overall material retention, but they do not by themselves distinguish between the retention of cellulose and hemicelluloses and the retention of residual lignin, extractives, or inorganic matter.

The nitrate–alkaline treatments produced yields of 31.57–36.83%, whereas the soda treatments produced yields of 45.18 and 47.66%, and the peracetic-acid treatment gave the highest yield of 55.68%. Because the chemical composition of the resulting pulps was not determined, the high yield of the peracetic-acid variant cannot be attributed directly to better carbohydrate preservation. Quantitative analysis of cellulose, hemicelluloses, acid-insoluble and acid-soluble lignin, extractives, and ash in each pulp would be required to establish component-specific retention. Detailed chemical and structural characterization is particularly important for lignin-containing cellulose materials, for which yield and a single lignin-related parameter cannot fully describe the composition or morphology [23].

The lower yields of the nitrate–alkaline variants may have resulted from the combined acidic and alkaline treatment stages and the associated dissolution of lignin and carbohydrate components [17]. This explanation should nevertheless be regarded as a hypothesis until the dissolved fractions and the composition of the resulting pulps are analyzed.

Within the nitrate–alkaline treatments, variant 3, which used 25% NaOH, produced the lowest pulp yield and the weakest paper. These results are consistent with more extensive dissolution or structural alteration of the fibrous fraction at the higher alkali concentration. However, cellulose depolymerization and carbohydrate loss were not measured directly.

In contrast, nitrate–alkaline variant 4, treated with 5% NaOH, achieved a higher yield and the lowest Kappa number among the nitrate–alkaline variants. This indicates that, within this particular treatment sequence, increasing the NaOH concentration did not improve the overall balance between yield, delignification, and paper strength.

The reject content was low in all variants, with the maximum value of 0.705% observed for Soda 2. The higher NaOH concentration therefore did not result in completely homogeneous fiber separation during the 30 min treatment. The residual rejects may be associated with the heterogeneous anatomical structure of cereal straw, including nodes, vascular bundles, epidermal tissues, and locally lignified particles that respond differently to chemical treatment [24]. Because the reject fraction was determined only gravimetrically, its anatomical and chemical composition remains unknown.

The soda pulps had Kappa numbers of 47.86 and 32.50, compared with 16.49–22.14 for the nitrate–alkaline pulps and 13.50 for the peracetic-acid pulp. The relatively high Kappa numbers and low brightness values of the soda pulps should primarily be interpreted in relation to the intentionally mild 30 min soda treatment. The soda conditions were selected to compare two NaOH concentrations while limiting prolonged alkaline exposure of the carbohydrate fraction.

They therefore do not represent a fundamental limitation of soda pulping. Longer cooking times, higher temperatures, modified liquor-to-straw ratios, or optimized alkali charges could increase delignification and brightness, although such changes might also reduce pulp yield and carbohydrate retention. Consequently, the present results only demonstrate that the soda variants were insufficiently delignified under the applied laboratory conditions.

The peracetic-acid variant combined the lowest Kappa number (13.50) with the highest yield (55.68%) and the highest brightness (76.77% ISO). Peracetic acid acts as an electrophilic oxidizing agent and preferentially attacks electron-rich phenolic groups and conjugated structures in lignin. Oxidation and cleavage of these structures facilitate lignin fragmentation and solubilization while simultaneously destroying lignin-derived chromophores [19,20]. This mechanism provides a plausible explanation for the combination of low Kappa number and high brightness obtained without an additional bleaching stage.

Previous studies have also shown that peracetic acid can achieve extensive delignification under comparatively mild conditions, although the retention of individual carbohydrate fractions depends on reaction conditions and must be determined analytically [19,25]. Therefore, the present results suggest favorable selectivity under the tested conditions but do not independently demonstrate selective preservation of cellulose or hemicelluloses.

The experiment also does not establish whether this performance is unique to peracetic acid. Other oxidative agents, including hydrogen peroxide, chlorine dioxide, and ozone, may also provide effective delignification and chromophore removal under optimized conditions. A direct comparison at equivalent delignification levels or process severities would be required to distinguish the intrinsic selectivity of these systems.

The tear index varied from 0.58 to 6.26 mN·m^2^·g^−1^ and the burst index from 0.23 to 0.67 kPa·m^2^·g^−1^, although the degree of beating varied within the relatively narrow range of 11.05–17.61 °SR. This indicates that the variation in mechanical properties cannot be explained by beating degree alone.

The high tear index of the peracetic-acid pulp suggests that fiber length and integrity were better preserved than in the more severe nitrate–alkaline treatments. Its comparatively high burst index also indicates effective inter-fiber bonding. The microscopic images support this interpretation, as the peracetic-acid sheet formed the most continuous and homogeneous network, with fewer coarse bundles and structural discontinuities. Studies of wheat-straw fibers used to reinforce recycled paper have similarly shown that suitable chemical treatment can increase fiber accessibility and improve the mechanical performance of the resulting sheet [24].

One-way ANOVA followed by Tukey’s HSD test identified significant differences among the seven pulp variants for both tear index (F(6,14) = 4640.1, *p* < 0.001) and burst index (F(6,14) = 1151.0, *p* < 0.001). However, only one pulping batch was prepared for each variant, and the tested sheets therefore represent sheet-level technical replicates rather than independent process replicates. The statistical results quantify within-batch differences among the examined pulp samples but cannot be generalized to between-batch process variability.

The high tear and burst indices of the peracetic-acid pulp are consistent with the presence of a comparatively well-bonded fiber network and possibly with better retention of load-bearing fibers. Nevertheless, fiber-length distribution, fines content, sheet density, porosity, and bonding area were not measured quantitatively. The optical micrographs provide qualitative evidence of fiber separation and network uniformity but cannot confirm the mechanisms responsible for the observed strength differences.

Accordingly, interpretations based on fiber-length preservation, fines generation, and inter-fiber bonding should be regarded as plausible explanations rather than directly demonstrated mechanisms [24,25,26]. Quantitative fiber analysis and measurements of sheet density and porosity would be necessary to test these hypotheses.

The relatively high degree of beating of nitrate–alkaline variant 3 did not result in high tear or burst strength. This may reflect differences in fiber swelling, flexibility, external fibrillation, or the presence of fine material; however, fines content was not measured. The Schopper–Riegler value is influenced by several pulp characteristics and cannot be interpreted as a direct measurement of fiber shortening or fibrillation.

Because cereal-straw fibers are generally shorter and more heterogeneous than softwood fibers, their most realistic use may involve blending with other pulps or application in paper grades that do not require the long-fiber reinforcement characteristic of softwood kraft pulp [25,26,27].

The peracetic-acid pulp reached a brightness of 76.77% ISO without an additional bleaching treatment. The corresponding ANOVA demonstrated significant differences among treatments (F(6,28) = 394.16, *p* < 0.001). The result is consistent with simultaneous lignin removal and oxidative destruction of chromophoric groups by peracetic acid [19,20].

The soda pulps showed substantially lower brightness values of 28.77–33.23% ISO. As noted above, these values should not be regarded as an intrinsic optical limitation of soda pulping because the soda treatments were comparatively mild and were not optimized to produce fully delignified or bleachable-grade pulp. The observation that Soda 2 had a lower Kappa number but did not exhibit proportionally higher brightness also confirms that brightness is influenced by chromophore chemistry and not exclusively by total residual lignin content.

The Cobb_60_ values ranged from 16.77 to 41.67 g·m^−2^. Significant differences among variants were detected by ANOVA (F(6,7) = 52.886, *p* < 0.001), although only two sheet measurements were available for each variant and the results should therefore be interpreted cautiously.

The lower water absorptiveness of the soda pulps was caused by their higher residual lignin content, which reduced surface wettability. The relatively low Cobb_60_ values of the soda pulps may be related to the lower wettability associated with residual lignin, but sheet density, pore structure, surface roughness, and capillary pathways may also have contributed. Conversely, more extensive delignification may expose additional hydrophilic polysaccharide surfaces without necessarily increasing water uptake if the resulting sheet structure is dense and well bonded.

The intermediate Cobb_60_ value of the peracetic-acid paper may therefore reflect a combination of surface chemistry and sheet structure. Because contact angle, liquid penetration kinetics, density, and porosity were not measured, the present data cannot distinguish among these mechanisms. Contact-angle and dynamic wettability measurements should be included in future work to verify the proposed relationship between residual lignin and water absorption [28].

The three processes produced pulps with different potential application profiles. Under the tested conditions, the dark soda pulps would be most relevant to unbleached packaging papers, inner layers of multilayer boards, molded-fiber products, or blends in which high optical quality is not required. The nitrate–alkaline pulps achieved lower Kappa numbers but were limited by their comparatively low yields and, particularly for variant 3, low mechanical performance. The peracetic-acid pulp appears most promising for lighter-colored packaging, specialty non-wood papers, or as a component of blended furnishes in which both brightness and tear resistance are required. Its brightness of 76.77% ISO was achieved without additional bleaching, although further treatment would probably be required for high-brightness printing and graphic grades.

Direct comparison with commercial wood pulps is complicated by differences in refining, basis weight, sheet preparation, and test conditions. As an illustrative comparison, wheat-straw paper refined to 28–56 °SR has been reported to achieve burst indices of approximately 1.2–2.8 kPa·m^2^·g^−1^ and tear indices of approximately 2.8–3.3 mN·m^2^·g^−1^, with the material proposed as a base paper for barrier-coated packaging [28]. The peracetic-acid paper in the present study showed a higher tear index of 6.26 mN·m^2^·g^−1^ but a lower burst index of 0.67 kPa·m^2^·g^−1^ at a substantially lower beating degree. These comparisons are indicative rather than definitive and should be validated through application-specific trials, including refining optimization, sizing, coating, printing, and converting tests.

## 5. Conclusions

Barley straw was demonstrated to be a potentially suitable non-wood raw material for the preparation of papermaking pulp, although the quality of the resulting pulp and laboratory sheets depended strongly on the applied delignification process. The three tested routes differed markedly in pulp yield, residual lignin content, mechanical properties, brightness, and water absorptiveness. Because the treatment differed in reaction mechanism, chemical charge, number of stages, and treatment time, the results represent a comparison of the specific laboratory variants under the applied conditions rather than a comparison at equivalent process severity.

The nitrate–alkaline treatments achieved relatively low Kappa numbers, but they were accompanied by the lowest pulp yields and, in some variants, poor mechanical performance. Soda pulping provided higher yields, but the relatively mild 30 min treatments resulted in high Kappa numbers and low sheet brightness. Among the tested variants, oxidative peracetic-acid delignification gave the most favorable combination of properties. This treatment produced the highest pulp yield of 55.68%, the lowest Kappa number of 13.50, a low reject content, and the highest tear index, burst index, and ISO brightness. The resulting sheets also showed a comparatively uniform fiber network and an intermediate Cobb_60_ value. The statistical analysis confirmed significant sheet-level differences among the variants; however, because only one pulping batch was prepared for each treatment, these results describe the examined batches and do not quantify between-batch process variability.

These findings indicate that, under the tested conditions, peracetic-acid delignification combined efficient lignin removal with high overall material retention and was therefore the most promising of the examined variants for converting barley straw into papermaking pulp. However, because the chemical composition of the resulting pulps was not determined, the high yield cannot be attributed directly to improved cellulose or hemicellulose preservation. Similarly, the mechanisms proposed to explain the mechanical properties and water absorptiveness could not be confirmed because fiber-length distribution, fines content, sheet density, porosity, and contact angle were not measured.

Further research should focus on independent replication and optimization of chemical consumption and treatment time, quantitative evaluation of fiber morphology and carbohydrate retention, direct characterization of sheet structure and surface wettability, and testing in specific applications such as packaging and blended paper furnishes. Chemical stability, corrosion, wastewater composition, acetic-acid and peroxide recovery, process economics, and environmental impacts must be evaluated before the industrial competitiveness of the peracetic-acid process can be established.

## Figures and Tables

**Table 1:** Experimental conditions used for the preparation of barley pulps.

Delignification Process	Treatment Conditions	Temperature	Treatment Time
Nitrate–alkaline 1	6% HNO_3_, 10% NaOH	101.3	30 + 30 min
Nitrate–alkaline 2	6% HNO_3_, 15% NaOH	100.9	30 + 30 min
Nitrate–alkaline 3	6% HNO_3_, 25% NaOH	104.2	30 + 30 min
Nitrate–alkaline 4	6% HNO_3_, 5% NaOH	102.6	30 + 30 min
Soda 1	15% NaOH	101.7	30 min
Soda 2	25% NaOH	105.2	30 min
Peracetic-acid	8% peracetic acid and 6% residual H_2_O_2_	95.0	45 min

**Table 2:** Chemical composition of the raw barley straw.

Material	Ash, %	Extractives, %	Acid-Insoluble Lignin, %	Cellulose, %	Hemicelluloses, %
Barley straw	7.65 ± 0.77	4.22 ± 0.28	18.79 ± 0.61	35.84 ± 0.43	40.77 ± 1.39

Values are presented as mean ± standard deviation (*n* = 3). Hemicellulose content was calculated as the difference between holocellulose and Seifert cellulose. Therefore, hemicellulose represents an estimated rather than directly measured fraction. Acid-soluble lignin was not determined.

**Table 3:** General properties of pulps prepared using different delignification treatments.

Pulp	Total Yield, %	Reject Content, %	Degree of Beating, SR	Kappa Number
Nitrate–alkaline 1	33.38	0.069	11.05 ± 0.14	17.86 ± 0.56
Nitrate–alkaline 2	32.36	0.128	17.61 ± 0.37	22.14 ± 0.93
Nitrate–alkaline 3	31.57	0.031	15.84 ± 0.62	18.49 ± 0.96
Nitrate–alkaline 4	36.83	0.193	12.45 ± 0.86	16.49 ± 0.48
Soda 1	45.18	0.217	15.17 ± 1.27	47.86 ± 1.43
Soda 2	47.66	0.705	15.83 ± 0.62	32.50 ± 1.66
Peracetic-acid	55.68	0.043	14.93 ± 0.16	13.50 ± 1.09

**Table 4:** Mechanical, optical, and physical properties of laboratory paper sheets prepared from barley-straw pulps.

Pulp	Tear Index, mN·m^2^·g^−1^	Burst Index, kPa·m^2^·g^−1^	ISO Brightness, %	Cobb_60_, g·m^−2^
Nitrate–alkaline 1	4.03 ± 0.05 ^b^	0.29 ± 0.00 ^b^	53.81 ± 0.16 ^bc^	26.50 ± 2.88 ^b^
Nitrate–alkaline 2	2.73 ± 0.03 ^d^	0.27 ± 0.00 ^bc^	50.62 ± 0.32 ^cd^	26.50 ± 1.63 ^b^
Nitrate–alkaline 3	0.58 ± 0.01 ^f^	0.23 ± 0.00 ^d^	48.59 ± 4.19 ^d^	41.67 ± 3.29 ^a^
Nitrate–alkaline 4	2.53 ± 0.03 ^c^	0.25 ± 0.00 ^d^	54.23 ± 1.50 ^e^	40.92 ± 0.24 ^c^
Soda 1	3.46 ± 0.04 ^c^	0.24 ± 0.01 ^d^	33.23 ± 0.09 ^e^	16.77 ± 0.74 ^c^
Soda 2	2.48 ± 0.03 ^e^	0.28 ± 0.00 ^b^	28.77 ± 0.27 ^f^	17.06 ± 0.87 ^c^
Peracetic-acid	6.26 ± 0.08 ^a^	0.67 ± 0.02 ^a^	76.77 ± 1.38 ^a^	23.45 ± 2.09

Values are presented as mean ± standard deviation (*n* = 10). Different superscript letters within the same column indicate significant differences among sheet-level measurements according to Tukey’s HSD test (*p* < 0.05).

## Data Availability

The data presented in this study are available on request from the corresponding author due to internal project.
